# Epilepsy and attention-deficit/hyperactivity disorder in children and adolescents: An overview of etiology, prevalence, and treatment

**DOI:** 10.3389/fnhum.2023.1021605

**Published:** 2023-04-11

**Authors:** Shimrit Uliel-Sibony, Veronika Chernuha, Itay Tokatly Latzer, Yael Leitner

**Affiliations:** ^1^Pediatric Neurology Institute, Dana-Dwek Children’s Hospital, Tel Aviv, Israel; ^2^The Pediatric ADHD Clinic, Tel Aviv Sourasky Medical Center Affiliated to the Sackler Faculty of Medicine, Tel Aviv University, Tel Aviv, Israel

**Keywords:** epilepsy, attention-deficit/hyperactivity disorder, children, adolescents, treatment, etiology

## Abstract

Epilepsy and attention-deficit/hyperactivity disorder (ADHD) are closely connected and commonly seen in both children and adults. Each of the disorders has major psychosocial and quality of life (QOL) effects, and their co-occurrence makes coping even more challenging for both the patients and their families. Moreover, an adverse effect of some anti-seizure medications can potentially induce or exacerbate symptoms of ADHD on the one hand, while some ADHD medications may increase seizure risk on the other. The combination of proper diagnosis and appropriate treatment may improve or even prevent many of the complications associated with these conditions. The objectives of this review are to present the complex relationship between epilepsy and ADHD, looking into the pathophysiological, anatomical, and functional perspectives along with the psychosocial and QOL influences and the recommended treatment approaches in accordance with the latest literature.

## Introduction

Attention-deficit/hyperactivity disorder (ADHD) is one of the most common neurobehavioral disorders, with a prevalence of 5–7% among children and young adults (Sayal et al., 2018). It is also one of the most common comorbidities in children with epilepsy (Costa et al., 2015). Various population studies reported the prevalence rate of ADHD as being 2.7–4 times higher in patients diagnosed with epilepsy in comparison to the general population (Chou et al., 2013; Cohen et al., 2013; Chidi et al., 2014; Subchartanan et al., 2015; Bertelsen et al., 2016; Cervi et al., 2018; Choudhary et al., 2018; Wang et al., 2020). At the same time, a higher incidence of epilepsy was reported among children with ADHD than among children without ADHD (Andrade, 2020) when ADHD symptoms and diagnosis preceded the presentation of those of epilepsy. Hermann et al. (2007) studied 75 children (age 8–18 years) with new/recent-onset idiopathic epilepsy and 62 healthy controls. Those authors found that the ADHD symptom onset, along with many psychosocial complications preceded the diagnosis of epilepsy in 19/23 (82%) of the children in the epilepsy and ADHD group. Similarly, a population-based case-control study of all newly diagnosed unprovoked seizures among Icelandic children younger than 16 years found that ADHD was 2.5-fold more common among children with newly diagnosed seizures than in control subjects, indicating that both of these conditions may represent an epiphenomenon of underlying neurobiological abnormalities (Hesdorffer et al., 2004). The bidirectional correlation between ADHD and epilepsy supports a multifactorial etiology, possibly sharing a common pathophysiological path that connects those two pathologies (Chou et al., 2013).

Patients with coexisting epilepsy and ADHD present several different characteristics compared to patients with a sole diagnosis. The first is the disappearance of the usual sex differences in the prevalence of ADHD: in the absence of epilepsy, ADHD is seen in school-aged children with a male-to-female ratio of 2–3:1 while that ratio is 1:1 when coexisting with epilepsy (Socanski et al., 2013). A variation was also found in the prevalence of ADHD subtypes when the two diagnoses appeared in combination: there was a higher proportion of children with ADHD predominantly of the inattentive type, in comparison to the general population where the ADHD–combined subtype is the most frequent.

Many hypotheses have been proposed regarding the association between epilepsy and ADHD; the independent (circumstantial) hypothesis - holds that both disorders are highly prevalent, hence it is very probable that some of the children will eventually be found to have both diagnoses. The second theory claims dependent association which can be direct contribution of the seizures, interictal epileptiform discharges and the anti-seizure medications to the attention deficit and hyperactive behavior or a multifactorial linkage (Ahmed et al., 2022). The functional impairment of this network may be caused by a combination of genetic, environmental, and physiological factors which differ in their impact, eventually leading to a net result of hyperactivity, inattention, and impulsivity. All may affect brain connectivity, plasticity, apoptosis, and modify cognitive and behavioral parameters, even before the seizures appear.

We present a review of the possible pathophysiological and extrinsic factors which promote the disproportionately higher rate of ADHD among children with epilepsy.

### Methods

The records were obtained by searching the PubMed database for studies conducted in children and adolescents. Search terms were “Epilepsy” and “ADHD” or “Attention-Deficit/Hyperactivity Disorder” and “children” and “adolescents” and “pediatric age” with “anti-seizure treatment,” “epilepsy syndrome,” “interictal discharges,” “ADHD treatment,” “QOL,” “psychosocial,”, “etiology” and “pathophysiology.”

## Etiology and pathophysiology

### Genetics

The overlapping diagnosis of both conditions may be attributed to a common genetic etiology. Gonzalez-Heydrich et al.’s (2009) pilot study demonstrated a higher prevalence of ADHD in mothers of children with ADHD and epilepsy. Moreover, mothers demonstrated a higher rate of current ADHD symptoms were more likely to have a family history of epilepsy in first- or second-degree relatives (in addition to the index child). A nationwide Swedish cohort study reported that the children of mothers with epilepsy also had a significantly increased risk of ADHD (odds ratio [OR] 1.85, 95% confidence interval [CI] 1.75–1.86), as did the children of fathers with epilepsy (OR 1.64, 95% CI 1.54–1.74) and individuals whose siblings had epilepsy (OR 1.56, 95% CI 1.46–1.67) (Brikell et al., 2016). These findings suggest that ADHD symptoms in children with epilepsy and their first-degree family members reflect a shared genetic and/or environmental source.

### Anatomical and functional brain pathology

The latest technological abilities, such as quantitative and functional magnetic resonance imaging (MRI) point at another possible pathophysiology underlying the co-occurrence of epilepsy and ADHD. One study on MRI-based volumetric measurements demonstrated that ADHD and epilepsy were associated with a significantly increased volume of gray matter distributed throughout regions of the frontal lobe as well as with a smaller brainstem volume (Hermann et al., 2007). One possible explanation was that the neurodevelopmental processes of cortical pruning and increasing myelination with concomitant declines in cerebral gray and increasing cerebral white matter volumes in normally developing children is static or attenuated in children with epilepsy and ADHD, leading to the morphometric abnormality. The volumetric changes may also be an anatomical reflection of the frontal lobe dysfunction in ADHD (Pasini et al., 2007) combined with genetic or acquired circumstances leading to overgrowth of pathological neuronal networks, and hyperexcitability.

Nevertheless another study found reduced cortical thickness in the bilateral areas of the frontal, parietal, and temporal lobes (Saute et al., 2014). The findings of Saute et al. (2014) also indicated minor or no group differences in the metrics of the cortical surface area, volume, or curvature, and smaller subcortical volumes of the caudate, thalamus, hippocampus, and brainstem in children with epilepsy and ADHD compared to the healthy control group. These anatomic abnormalities were evident early in the course of epilepsy, suggesting the presence of antecedent neurodevelopmental changes.

The reported volumetric differences are not reflected in functional studies. Bechtel et al. (2012) recorded brain activation while performing working memory tasks in boys aged 8–14 years, seventeen of whom were diagnosed with combined epilepsy/ADHD, fifteen with developmental ADHD, and fifteen healthy controls. Those authors reported that both patient groups showed similar reductions of activation in all parts relevant to the functional network of working memory compared to the control group, suggesting that ADHD with and without epilepsy share a common underlying neurobehavioral pathophysiology (although no children with epilepsy without ADHD were included in either study for comparison) (Bechtel et al., 2012).

Despite the disparate methodologies of these studies, the conclusions are in keeping with those reported in studies of idiopathic ADHD, which have shown disruption within a broad fronto-striatal-cerebellar network (De La Fuente et al., 2013) and similar patterns of cortical changes (Narr et al., 2009). Therefore whether a unique anatomical/morphological is phenotype of epilepsy-associated ADHD is still controversial (Yoong, 2015).

There is also a neurotransmitter-related hypothesis based upon animal models of ADHD, which suggests that functional impairments in excitatory glutamatergic synaptic transmissions may be one of the underlying mechanisms leading to increased susceptibility to both epilepsy and ADHD (Jensen et al., 2009).

### Epilepsy syndrome, onset, type, and seizure load as related to coexisting ADHD

There are certain generalized epileptic and non-lesional focal syndromes which are more likely to present with ADHD-like behaviors, supporting the structural/genetic theory. Self-limited epilepsy with centrotemporal spikes (SeLECTS) is probably the epileptic syndrome most identified with ADHD. The frequency of centrotemporal spikes in non-epileptic children with ADHD is significantly higher than would be expected in these children (Holtmann et al., 2003; Aricò et al., 2020). When pediatric patients diagnosed with SeLECTS were specifically tested for distractors in their visual field, impaired attentional control was detected even in comparison to patients with childhood absence epilepsy (CAE) (Deltour et al., 2007). Comparable results were found in frontal lobe epilepsy in a cohort study of non-lesional frontal lobe epilepsy: as many as one-third of the patients exhibited ADHD or impulsivity difficulties (Prévost et al., 2006). The prefrontal cortex pre-supplementary motor area and the inferior frontal gyrus (IFG), have a key role in the executive functions, such as inhibition control and set shifting The high prevalence of ADHD in patients with frontal lobe epilepsy may be a reflection of the dysregulation of the frontal lobe networks (Ahmed et al., 2022). The remarkable association of the frontal lobe and, as evidenced biologically, electrographically, and structurally, is also reflected in benign well-controlled CAE that as generalized epilepsy involves major frontal lobe networks. Caplan et al. (2008) reported a 26% rate of attention and behavioral deficit among children with CAE compared to 6% in the general population. Whether this is secondary to a genetic predisposition, an MRI-silent developmental lesion, or the repercussion of epileptic discharges on frontal lobe functions remains uncertain.

Regardless of the epilepsy type and anatomical location of seizures, there is robust evidence that uncontrolled epilepsy with a high seizure load is strongly associated with ADHD. McCusker et al. (2002) performed standardized measures of child and family adjustment among 48 families of children with intractable epilepsy and no associated severe learning disability, of whom the greatest behavioral problems were related to attentional and hyperactivity disturbances, who attended a regional pediatric neurology service. One of the two factors which were persistently implicated across a range of adjustment problems was the frequency of rectal diazepam administration. Another review of 75 children with epilepsy concluded that those with seizures occurring at least weekly were rated by their parents as having more symptoms of hyperactivity than those with less frequent seizures (Berl et al., 2015).

Young age at epilepsy onset is a significant neurodevelopmental risk factor, which is hypothesized to have a more detrimental effect on the immature developing brain than longer duration of the disease and multidrug treatment. Nevertheless, there is no consensus regarding the effect of young age at epilepsy onset and a higher prevalence of ADHD. Kwong et al. (2016) examined the association between ADHD and epilepsy in 122 adolescents, taking into consideration seizure-related and sociodemographic variables. Those authors reported a negative correlation between ADHD scores and age at seizure onset. Similarly, in a study of 21 patients with SeLECTS who were diagnosed with coexisting ADHD, those who were younger at the onset of epilepsy demonstrated a lower IQ, higher attention deficit (*p* = 0.004) and higher impulsivity (*p* = 0.016) (Danhofer et al., 2018). Those findings were not supported by the data published by Kral et al. (2016) who reviewed the neuropsychological evaluation records of 204 youths with epilepsy who were divided into groups of no ADHD, ADHD combined type, and ADHD predominantly inattentive type. In that study, the age of seizure onset, seizure classification, and seizure frequency did not differ significantly between the groups (Kral et al., 2016).

### Subclinical epileptiform activity

The relation or impact of interictal epileptiform discharges (IED) with attention and cognition in children has long been presumed. The prevalence of IED in the healthy population varied between 1.4 to 6.5% depending upon sex, age, state, and type of data collection (digital/non-digital) (Cavazzuti et al., 1980; Okubo et al., 1994; Capdevila et al., 2008; Borusiak et al., 2010). A similar prevalence of IED was detected in a population of children with ADHD who were evaluated in an alertness state (Socanski et al., 2015). However, when 42 children with ADHD underwent overnight electroencephalogram (EEG) recordings, 53% produced IED (Silvestri et al., 2007), suggesting that ADHD is a condition that is associated with EEG epileptiform abnormalities and that it plays a role in cognitive-behavioral abilities. Furthermore, Abd El Naby and Naguib (2018) suggested that IED’s specifically over frontal lobe, are involved in the pathophysiology of the combination of ADHD and epilepsy.

Interictal epileptiform discharges are known to contribute to behavioral issues in patients with epilepsy and other neurological disorders. The most persuasive support for the association of IED during sleep and the parameters of attention, behavior, and cognition (mainly language processing) is based upon studies in children with SeLECTS. This specific population has no seizures or a low seizure load, does not require anti-seizure medications (ASM), and awake IED may be rare compared to marked activation during sleep. In a review of the literature, Nicolai et al. (2006) concluded that the average results are reportedly lower for some executive functions and for sustained attention in the presence of other learning and memory difficulties in cases of uncomplicated SeLECTS compared with controls. Moreover, those authors found strong evidence that the problems in attention are related to right-sided discharges and the high rate of epileptiform discharges in sleep. A bilateral IED’s activity was found to be significantly more common in children with behavioral disorders than children without behavioral disorders (*p* = 0.039) in children with SeLECTS (Özgen et al., 2021). There is further support derived from neurological disorders without epilepsy. For example, Mulligan and Trauner (2014) identified 101 children with autism who had undergone 24-h EEGs: 50% had epileptiform abnormalities after children with a history of epilepsy had been excluded. The presence of epileptiform activity was associated with lower functional levels (intellectual and behavioral) in the children with autism, whereas the incidence of such abnormalities was only 20% among those with high-functioning autism. Another example is the relationship found between IEDs in twenty-three individuals with Smith-Lemli-Opitz and ADHD (Schreiber et al., 2014). In that study, 43 of 85 EEGs (51%) were abnormal. While IEDs group showed no significant between group differences in ADHD trait symptoms or epilepsy occurrence, the presence of single-visit IEDs predicted a 34% increase in hyperactive/impulsive symptom severity compared with visits without IEDs over time.

Two main hypotheses have been suggested in an attempt to explain the role of the interictal activity on behavior and cognition (Van Bogaert et al., 2012). The first theory is that IED activity causes a transients focal dysfunction of the cortical activity as was seen in a different evoked potential tests (Shewmon and Erwin, 1988) or that the IED case temporary alternation of a distal cortical and subcortical synaptic network which consequences on brain functioning evidenced cognitively and behaviorally (De Tiège et al., 2007; Siniatchkin et al., 2010). The second theory suggests a more long-lasting effect of the intense interictal activity during sleep causing cognitive impairment through dysfunction of both hypermetabolism at the site of the epileptic foci and hypometabolism in distant and connected brain areas (De Tiège et al., 2008, 2009). Another assumption is that memory consolidation processes are affected by the interfered sleep-dependent physiological processes of neuronal plasticity (Plihal and Born, 1999; Stickgold et al., 2000). IEDs may also represent a marker for an underlying process that impacts behavior/cognition may be proposed by the results of Capdevila et al. (2008). Among 4/6 healthy children who underwent polysomnographic test and IEDs were detected, abnormal findings in areas of behavior, attention, hyperactivity, and learning were diagnosed in a formal neurocognitive test.

### ADHD treatment

Attention-deficit/hyperactivity disorder is a disorder with a higher risk of seizures with a possibility that treatment itself may lower seizure threshold. Overall, there is a paucity of data about seizure risk in non-epileptic children treated with stimulants therefore there is no clear, evidence-based answer to this question. A cohort analysis evaluated the risk of seizures among pediatric patients naïve to ADHD medication therapy. No statistically significant difference was found in the adjusted relative risk in 13,322 patients exposed to atomoxetine relative to 13,398 patients treated with stimulant over a follow-up period of 6 months (McAfee et al., 2013). Similar results were published reviewing the Italian ADHD Registry where a total of 1,350 and 753 participants (aged 6–18 years) were treated with methylphenidate and atomoxetine, respectively. Over 5 years follow up seizures occurred in 3 patient in the methylphenidate group and two in the atomoxetine group (Cortese et al., 2015). When EEG preformed in 243 patients with ADHD prior to stimulant treatment initiation seizures occurred only in the treated group, in 1/175 patients with a normal EEG and 3/30 treated patients with epileptiform EEGs. As expected, epileptiform EEG in healthy children with ADHD predicts considerable risk of seizure occurrence with treatment. The risk, however, is not necessarily attributable to stimulant use (Hemmer et al., 2001).

### Psychosocial factors

Intrafamilial relations (for example, the degree of conflict/cohesion) were directly associated with adjustment difficulties in the child with ADHD as well as with the frequency of seizures. In a survey of 48 families, McCusker et al. (2002) reported that the family functioning variables were one of the major predictors of behavioral and adjustment difficulties. Behavioral problems and competencies in the child were shown to have a direct relationship to the degree of conflict (expressed anger, aggression, and conflict among family members) and an inverse relation to the degree of family cohesion (commitment, helpfulness, and support). There was a direct effect of neuropsychological function on academic achievement (not specific to ADHD) in 173 children with chronic epilepsy (Fastenau et al., 2004). A comprehensive neuropsychological battery revealed that neuropsychological deficits had a smaller impact upon the achievement on children growing up in supportive/organized homes compared with children growing up in unsupportive/disorganized homes. Thus, the subgroup of children with epilepsy (those with not only neuropsychological deficits but also disorganized/unsupportive home environments) is particularly at risk for adverse academic outcomes.

### Contribution of ASM

The ASM and their side effects have significant potential of influencing many aspects of behavior, cognition, and quality of life (Hessen et al., 2006). The associated cognitive and behavioral side effects depend upon the combination of various factors, including different vulnerabilities, metabolic profiles, existing cognitive problems, modes of action, doses, concentrations, rates of titration, ages at treatment onset, durations of treatment, and concomitant medications (Lagae, 2006). Moreover, in many studies behavioral and cognitive difficulties were reported with anti-seizure medication, however, whether they completely fulfilled the criteria of shorter attention span with or without hyperactive behavior is not always clear. Review of the available evidence in the literature classified the medication according to their mode of action (Verrotti et al., 2018) or by generation (Lagae, 2006; Aldenkamp et al., 2016). Phenobarbital is generally considered as being the most frequently reported medication to induce symptoms of ADHD, followed by topiramate and valproic acid (Verrotti et al., 2018). GABAergic drugs specifically influence vigilance and attentional processes (Lagae, 2006). Furthermore, combination of phenobarbital, phenytoin, and primidone with Methylphenidate, which blocks their metabolism, leads to a higher levels of these medications and a greater risk of side effects, especially on attentiveness. The anti-glutamatergic drugs, especially those that work on the NMDA receptor-like perampanel (though with low affinity), are more associated with negative cognition effects on learning and memory (Rosenfeld et al., 2015).

According to the International League Against Epilepsy (ILAE) task force 2016 report on the older group of medications, phenobarbital showed the most convincing evidence of negative effects on both cognition and behavior, with behavioral problems ranging from hyperactivity to withdrawal or sedation (Aldenkamp et al., 2016). Similarly, caution was recommended in prescribing valproic acid due to insufficient information to confirm the clinical impression that it is associated with behavioral complications. This recommendation was based upon Masur et al.’s (2013) study that provided Class I evidence that valproic acid is associated with more significant problems of attention than ethosuximide or lamotrigine in children with newly diagnosed CAE. That finding was based upon an age-specific battery of neuropsychological tests, as well as upon questionnaires on behavior and quality of life. Furthermore, prenatal exposure to valproic acid reportedly caused a high level of inattentiveness and hyperactivity in children at 3 years of age (Cohen et al., 2011). Clobazam, carbamazepine, and ethosuximide were found to be safe in terms of cognitive and behavioral effects in children, with well-established evidence in the literature for those first two medications and lack of either positive or negative evidence regarding the latter (Verrotti et al., 2018).

Among the newer ASM, the interpretation of the behavioral data for vigabatrin was distorted since the primary etiology of the majority of the studied children included those with behavioral impairments. Topiramate can cause adverse effects in behavior, particularly in children and adolescents with intellectual disabilities, and those effects tend to persist as long as the treatment is continued. This is also the case for levetiracetam, where the available data are still contradictory, although they suggest that caution should be used due to possible association with aggression (Levisohn et al., 2009; Chen et al., 2017). Oxcarbazepine, gabapentin, and felbamate either showed no evidence for a cognitive or a behavioral effect, or the available information was insufficient to establish their influence. Lamotrigine was the only medication found to have a positive impact on both cognition and behavior (Han et al., 2017). Information regarding zonisamide is limited, therefore careful behavioral and cognitive monitoring is recommended. Information on the cognitive impact of stiripentol, lacosamide, and rufinamide was inconclusive (Auvin et al., 2018; Verrotti et al., 2018). A pooled analysis derived from three Phase III trials and an open-label extension study found that 8.2% of 143 adolescents receiving perampanel showed aggressive behavior (Rosenfeld et al., 2015). It was suggested that a behavioral change, including aggression, might be dose-related but more data are required (Aldenkamp et al., 2016).

The evidence for behavior and cognitive difficulties contributed by anti-seizure treatment is more established than in related to the specific symptoms of ADHD. Therefore, any medication which may cause significant behavioral or cognitive side effects should be avoided or be used with caution, in patients with ADHD or those who already struggle with these challenges.

## Diagnosis

A consensus paper issued by the Task Force on Comorbidities of the ILAE Pediatric Commission in 2018 recommended screening for ADHD in every child with epilepsy starting at the age of 6 years, or at the time of diagnosis if older than 6 years. The child should be reevaluated annually and after any ASM change. The screening should not be performed within 48 h of a seizure associated with a postictal state (Auvin et al., 2018). It should be undertaken by health practitioners with expertise in ADHD, such as child psychiatrists, child neurologists, developmental pediatricians, and psychologists. Since intellectual disability and learning disabilities are common comorbidities of epilepsy, formal cognitive testing in children with epilepsy is recommended for those children struggling at school (Auvin et al., 2018).

## Treatment

The primary role and efficacy of the medications indicated for ADHD are well established in the literature and their use is common practice in pediatric neurology, including children with epilepsy (Rheims et al., 2016). However, concerns are often expressed by parents when a child with epilepsy requires ADHD treatment as well. Methylphenidate (MPH) is the most commonly prescribed medication for treating ADHD. It blocks the reuptake of dopamine and noradrenaline into neurons and appears to stimulate the cerebral cortex and subcortical structures (Volkow et al., 2002). Therefore, initial caution is warranted for using MPH in the presence of seizures. A retrospective study of 105 pediatric patients (mean age 14.8 years) with epilepsy who were treated with MPH for a mean duration of 22 months showed exacerbation of seizures in 20% of the subjects and worsened EEG findings in 32% of the subjects (Park et al., 2018). Moore et al. (2002) reported a 12.5% increase of mild transient seizures in a prospective study of eleven children and adults with epilepsy. The therapeutic approach of MPH has changed over time and as a result of additional clinical published studies. One of the first of those studies that appeared in 1997 was by Gross-Tsur et al. (1997) who followed 30 patients for 4 months and found MPH to be effective in treating children with epilepsy and ADHD and safer in children who were seizure-free later on. This was followed by a retrospective cohort study that compared 18,166 children with epilepsy who were being treated with stimulants and 54,197 non-users. No increase in seizure-related hospitalizations with the use of stimulants was detected in the children with epilepsy (Liu et al., 2018). Similarly, Adams et al. (2017) described a prospective double-blind, placebo-controlled study of 30 adult patients who did not show any significant change in seizure frequency but who experienced a moderate to large improvement in ADHD symptoms. Based upon that study, the ILAE task force of comorbidities in the pediatric population assigned it a level B evidence for efficacy, safety, and tolerability of MPH in children with epilepsy (Auvin et al., 2018).

There is limited data on other ADHD medications. Amphetamines were found to be less effective than MPH (24 vs. 63%, respectively) in an observational retrospective study of 36 children and adolescents. Seizure aggravation was not reported, however, the level of tolerability was low, with a dropout of 53% of amphetamine-treated patients due to side effects (Gonzalez-Heydrich et al., 2014). Atomoxetine showed limited efficacy (37–59%) in two observational clinically based studies (Torres et al., 2011; Auvin et al., 2018) with a seizure worsening rate of 7–9%. A recent retrospective study of 105 patients reported a significant decrease in the Clinical Global Impression Scale score over 12 weeks of atomoxetine treatment with a similar rate of seizure aggravation (Park et al., 2020). Brikell at al reviewed the risk of acute seizure Among 995 individuals with epilepsy, under the age of 19 who initiated ADHD medication (stimulants, atomoxetine, or both) in Sweden. During 24 weeks follow-up, within-individual analyses, no statistically significant difference was seen in the rate of seizures in comparison to the same period of time before medication initiation (Brikell et al., 2019). On a different perspective but further supporting of the safety of ADHD medication (stimulants, atomoxetine or both), Wiggs et al. (2018) demonstrated that ADHD treatment was associated with lower risk of seizures (29 and 49% lower odds in patients with history of seizures and with no recorded prior seizures, respectively), in relative to unmedicated periods. It is therefore sufficient to conclude that MPH may be safely used in children and adolescents with a current or past history of epilepsy.

## Summary

Attention-deficit/hyperactivity disorder is a ubiquitous and major developmental and functional challenge in children with epilepsy. The direct cause for this comorbidity has not yet been elucidated, although we do know that it is probably not a direct result of a single factor, but rather of many extrinsic and intrinsic components in combination. Each patient presents a variety of characteristics which eventually determine whether ADHD will become a significant comorbidity. Pediatric neurologists must be aware of this comorbidity, assess it early in the course of the disease, and determine whether it preceded the onset of epilepsy. Upon diagnosis, depending on severity, and alongside the medical treatment, we should consider treating or changing existing factors such as ASM, parental education, and seizure control. Future and more systematic studies on the association between epilepsy and ADHD are warranted with the aim of refining current approaches and enhancing the pediatric patient’s function, achievements, and behavior.

## Data availability statement

The original contributions presented in this study are included in the article/supplementary material, further inquiries can be directed to the corresponding author.

## Author contributions

SU-S, VC, IT, and YL contributed to the conception and design of the study. SU-S wrote the first draft of the manuscript. VC, IT, and YL wrote sections of the manuscript. All authors contributed to manuscript revision, read, and approved the submitted version.

## References

[B1] Abd El Naby S. Naguib Y. (2018). Sociodemographic, electrophysiological, and biochemical profiles in children with attention deficit hyperactivity disorder and/or epilepsy. *Behav. Neurol.* 2018:8932817. 10.1155/2018/8932817 30631381 PMC6305032

[B2] Adams J. Alipio-Jocson V. Inoyama K. Bartlett V. Sandhu S. Oso J. (2017). Methylphenidate, cognition, and epilepsy: A 1-month open-label trial. *Epilepsia* 58 2124–2132. 10.1111/epi.13917 28990169

[B3] Ahmed G. Metwaly N. Elbeh K. Galal M. Shaaban I. (2022). Prevalence of school bullying and its relationship with attention deficit-hyperactivity disorder and conduct disorder: A cross-sectional study. *Egypt J. Neurol. Psychiatr. Neurosurg.* 58:60. 10.1186/s41983-022-00494-6 35645553 PMC9125342

[B4] Aldenkamp A. Besag F. Gobbi G. Caplan R. Dunn D. W. Sillanpää M. (2016). Psychiatric and behavioural disorders in children with epilepsy (ILAE Task Force Report): Adverse cognitive and behavioural effects of antiepileptic drugs in children. *Epilept. Disord.* 18 S55–S67. 10.1684/epd.2016.0817 27210239

[B5] Andrade C. (2020). Methylphenidate and the risk of new-onset seizures. *J. Clin. Psychiatry* 81:20f13586. 10.4088/JCP.20f13586 32726522

[B6] Aricò M. Arigliani E. Giannotti F. Romani M. (2020). ADHD and ADHD-related neural networks in benign epilepsy with centrotemporal spikes: A systematic review. *Epilepsy Behav.* 112:107448. 10.1016/j.yebeh.2020.107448 32916583

[B7] Auvin S. Wirrell E. Donald K. Berl M. Hartmann H. Valente K. (2018). Systematic review of the screening, diagnosis, and management of ADHD in children with epilepsy. Consensus paper of the task force on comorbidities of the ILAE pediatric commission. *Epilepsia* 59 1867–1880. 10.1111/epi.14549 30178479

[B8] Bechtel N. Kobel M. Penner I. Specht K. Klarhöfer M. Scheffler K. (2012). Attention-deficit/hyperactivity disorder in childhood epilepsy: A neuropsychological and functional imaging study. *Epilepsia* 53 325–333. 10.1111/j.1528-1167.2011.03377.x 22242637

[B9] Berl M. Terwilliger V. Scheller A. Sepeta L. Walkowiak J. Gaillard W. (2015). Speed and complexity characterize attention problems in children with localization-related epilepsy. *Epilepsia* 56 833–840. 10.1111/epi.12985 25940056 PMC4457628

[B10] Bertelsen E. Larsen J. Petersen L. Christensen J. Dalsgaard S. (2016). Childhood epilepsy, febrile seizures, and subsequent risk of ADHD. *Pediatrics* 138:e20154654. 10.1542/peds.2015-4654 27412639

[B11] Borusiak P. Zilbauer M. Jenke A. (2010). Prevalence of epileptiform discharges in healthy children–new data from a prospective study using digital EEG. *Epilepsia* 51 1185–1188. 10.1111/j.1528-1167.2009.02411.x 20002145

[B12] Brikell I. Chen Q. Kuja-Halkola R. D’Onofrio B. Wiggs K. Lichtenstein P. (2019). Medication treatment for attention-deficit/hyperactivity disorder and the risk of acute seizures in individuals with epilepsy. *Epilepsia* 60 284–293. 10.1111/epi.14640 30682219 PMC6365170

[B13] Brikell I. Kuja-Halkola R. D’Onofrio B. (2016). Familial liability of ADHD and epilepsy: A nationwide cohort study. *Behav. Genet.* 46:774. 10.1016/j.biopsych.2017.08.006 28950988 PMC5723535

[B14] Capdevila O. Dayyat E. Kheirandish-Gozal L. Gozal D. (2008). Prevalence of epileptiform activity in healthy children during sleep. *Sleep Med.* 9 303–309. 10.1016/j.sleep.2007.03.024 17638587

[B15] Caplan R. Siddarth P. Stahl L. Lanphier E. Vona P. Gurbani S. (2008). Childhood absence epilepsy: Behavioral, cognitive, and linguistic comorbidities. *Epilepsia* 49 1838–1846. 10.1111/j.1528-1167.2008.01680.x 18557780

[B16] Cavazzuti G. Cappella L. Nalin A. (1980). Longitudinal study of epileptiform EEG patterns in normal children. *Epilepsia* 21 43–55. 10.1111/j.1528-1157.1980.tb04043.x 6766393

[B17] Cervi F. Silvestri M. Turner K. Cavirani B. Mingarelli A. Caputo D. (2018). Assessment of adhd in a cohort of children and adolescents with epilepsy. *Epilepsia* 59:S50.

[B18] Chen B. Detyniecki K. Choi H. Hirsch L. Katz A. Legge A. (2017). Psychiatric and behavioral side effects of anti-epileptic drugs in adolescents and children with epilepsy. *Eur. J. Paediatr. Neurol.* 21 441–449. 10.1016/j.ejpn.2017.02.003 28238621

[B19] Chidi I. Chidi N. Ebele A. Chinyelu O. (2014). Co-Morbidity of attention deficit Hyperactivity Disorder (ADHD) and epilepsy In children seen In University of Nigeria Teaching Hospital Enugu: Prevalence, clinical and social correlates. *Niger Postgrad. Med. J.* 21 273–278. 25633443

[B20] Chou I. Chang Y. Chin Z. Muo C. Sung F. Kuo H. (2013). Correlation between epilepsy and attention deficit hyperactivity disorder: A population-based cohort study. *PLoS One* 8:e57926. 10.1371/journal.pone.0057926 23483944 PMC3590288

[B21] Choudhary A. Gulati S. Sagar R. Sankhyan N. Sripada K. (2018). Childhood epilepsy and ADHD comorbidity in an Indian tertiary medical center outpatient population. *Sci. Rep.* 8:2670. 10.1038/s41598-018-20676-8 29422636 PMC5805699

[B22] Cohen M. Meador K. Browning N. Baker G. Clayton-Smith J. Kalayjian L. (2011). Fetal antiepileptic drug exposure: Motor, adaptive, and emotional/behavioral functioning at age 3 years. *Epilepsy Behav.* 22 240–246. 10.1016/j.yebeh.2011.06.014 21783425 PMC3185140

[B23] Cohen R. Senecky Y. Shuper A. Inbar D. Chodick G. Shalev V. (2013). Prevalence of epilepsy and attention-deficit hyperactivity (ADHD) disorder: A population-based study. *J. Child Neurol.* 28 120–123. 10.1177/0883073812440327 22550087

[B24] Cortese S. Panei P. Arcieri R. Germinario E. Capuano A. Margari L. (2015). Safety of methylphenidate and atomoxetine in children with attention-deficit/hyperactivity disorder (ADHD): Data from the Italian national ADHD registry. *CNS Drugs* 29 865–877. 10.1007/s40263-015-0266-7 26293742

[B25] Costa CR. Oliveira Gde M. Gomes Mda M Maia Filho Hde S. (2015). Clinical and neuropsychological assessment of attention and ADHD comorbidity in a sample of children and adolescents with idiopathic epilepsy. *Arq. Neuropsiquiatr.* 73 96–103. 10.1590/0004-282X20140219 25742577

[B26] Danhofer P. Pejèochová J. Dušek L. Rektor I. Ošlejšková H. (2018). The influence of EEG-detected nocturnal centrotemporal discharges on the expression of core symptoms of ADHD in children with benign childhood epilepsy with centrotemporal spikes (BCECTS): A prospective study in a tertiary referral center. *Epilepsy Behav.* 79 75–81. 10.1016/j.yebeh.2017.11.007 29253678

[B27] De La Fuente A. Xia S. Branch C. Li X. (2013). A review of attention-deficit/hyperactivity disorder from the perspective of brain networks. *Front. Hum. Neurosci.* 7:192. 10.3389/fnhum.2013.00192 23720619 PMC3654209

[B28] De Tiège X. Goldman S. Van Bogaert P. (2009). Insights into the pathophysiology of psychomotor regression in CSWS syndromes from FDG-PET and EEG-fMRI. *Epilepsia* 50 47–50. 10.1111/j.1528-1167.2009.02219.x 19682051

[B29] De Tiège X. Harrison S. Laufs H. Boyd S. Clark C. Allen P. (2007). Impact of interictal epileptic activity on normal brain function in epileptic encephalopathy: An electroencephalography-functional magnetic resonance imaging study. *Epilepsy Behav.* 11 460–465. 10.1016/j.yebeh.2007.06.001 17869185

[B30] De Tiège X. Ligot N. Goldman S. Poznanski N. de Saint Martin A. Van Bogaert P. (2008). Metabolic evidence for remote inhibition in epilepsies with continuous spike-waves during sleep. *Neuroimage* 40 802–810. 10.1016/j.neuroimage.2007.11.043 18201907

[B31] Deltour L. Barathon M. Quaglino V. Vernier M. Despretz P. Boucart M. (2007). Children with benign epilepsy with centrotemporal spikes (BECTS) show impaired attentional control: Evidence from an attentional capture paradigm. *Epilept. Disord.* 9 32–38. 10.1684/epd.2007.0066 17307709

[B32] Fastenau P. Shen J. Dunn D. Perkins S. Hermann B. Austin J. (2004). Neuropsychological predictors of academic underachievement in pediatric epilepsy: Moderating roles of demographic, seizure, and psychosocial variables. *Epilepsia* 45 1261–1272. 10.1111/j.0013-9580.2004.15204.x 15461681 PMC2736953

[B33] Gonzalez-Heydrich J. Hsin O. Gumlak S. Kimball K. Rober A. Azeem M. (2014). Comparing stimulant effects in youth with ADHD symptoms and epilepsy. *Epilepsy Behav.* 36 102–107. 10.1016/j.yebeh.2014.04.026 24907495 PMC4109643

[B34] Gonzalez-Heydrich J. Luna L. Rao S. McClendon J. Rotella P. Waber D. (2009). Elevated ADHD rates in mothers of children with comorbid ADHD and epilepsy. *Epilepsia* 50:236. 10.2217/npy.12.53 23397446 PMC3565178

[B35] Gross-Tsur V. Manor O. van der Meere J. Joseph A. Shalev R. (1997). Epilepsy and attention deficit hyperactivity disorder: Is methylphenidate safe and effective? *J. Pediatr.* 130 670–674. 10.1016/s0022-3476(97)70258-0 9432523

[B36] Han S. Yang E. Song M. Kim S. (2017). Effects of lamotrigine on attention-deficit hyperactivity disorder in pediatric epilepsy patients. *Korean J. Pediatr.* 60 189–195. 10.3345/kjp.2017.60.6.189 28690646 PMC5500387

[B37] Hemmer S. Pasternak J. Zecker S. Trommer B. (2001). Stimulant therapy and seizure risk in children with ADHD. *Pediatr. Neurol.* 24 99–102. 10.1016/s0887-8994(00)00240-x 11275457

[B38] Hermann B. Jones J. Dabbs K. Allen C. Sheth R. Fine J. (2007). The frequency, complications and aetiology of ADHD in new onset paediatric epilepsy. *Brain* 130 3135–3148. 10.1093/brain/awm227 17947336

[B39] Hesdorffer D. Ludvigsson P. Olafsson E. Gudmundsson G. Kjartansson O. Hauser W. (2004). ADHD as a risk factor for incident unprovoked seizures and epilepsy in children. *Arch. Gen. Psychiatry* 61 731–736. 10.1001/archpsyc.61.7.731 15237085

[B40] Hessen E. Lossius M. Reinvang I. Gjerstad L. (2006). Influence of major antiepileptic drugs on attention, reaction time, and speed of information processing: Results from a randomized, double-blind, placebo-controlled withdrawal study of seizure-free epilepsy patients receiving monotherapy. *Epilepsia* 47 2038–2045. 10.1111/j.1528-1167.2006.00805.x 17201701

[B41] Holtmann M. Becker K. Kentner-Figura B. Schmidt M. (2003). Increased frequency of rolandic spikes in ADHD children. *Epilepsia* 44 1241–1244. 10.1046/j.1528-1157.2003.13403.x 12919398

[B42] Jensen V. Rinholm J. Johansen T. Medin T. Storm-Mathisen J. Sagvolden T. (2009). N-methyl-D-aspartate receptor subunit dysfunction at hippocampal glutamatergic synapses in an animal model of attention-deficit/hyperactivity disorder. *Neuroscience* 158 353–364. 10.1016/j.neuroscience.2008.05.016 18571865

[B43] Kral M. Lally M. Boan A. (2016). Identification of ADHD in youth with epilepsy. *J. Pediatr. Rehabil. Med.* 9 223–229. 10.3233/PRM-160383 27612082

[B44] Kwong K. Lam D. Tsui S. Ngan M. Tsang B. Lam S. (2016). Attention deficit hyperactivity disorder in adolescents with epilepsy. *Pediatr. Neurol.* 57 56–63. 10.1016/j.pediatrneurol.2015.12.022 26831952

[B45] Lagae L. (2006). Cognitive side effects of anti-epileptic drugs. The relevance in childhood epilepsy. *Seizure* 15 235–241. 10.1016/j.seizure.2006.02.013 16563808

[B46] Levisohn P. Mintz M. Hunter S. Yang H. Jones J. (2009). Neurocognitive effects of adjunctive levetiracetam in children with partial-onset seizures: A randomized, double-blind, placebo-controlled, noninferiority trial. *Epilepsia* 50 2377–2389. 10.1111/j.1528-1167.2009.02197.x 19702752

[B47] Liu X. Carney P. Bussing R. Segal R. Cottler L. Winterstein A. (2018). Stimulants do not increase the risk of seizure-related hospitalizations in children with epilepsy. *J. Child Adolesc. Psychopharmacol.* 28 111–116. 10.1089/cap.2017.0110 29028437 PMC5911707

[B48] Masur D. Shinnar S. Cnaan A. Shinnar R. Clark P. Wang J. (2013). Pretreatment cognitive deficits and treatment effects on attention in childhood absence epilepsy. *Neurology* 81 1572–1580. 10.1212/WNL.0b013e3182a9f3ca 24089388 PMC3806916

[B49] McAfee A. Landon J. Jones M. Bangs M. Acharya N. Hornbuckle K. (2013). A cohort study of the risk of seizures in a pediatric population treated with atomoxetine or stimulant medications. *Pharmacoepidemiol. Drug Saf.* 22 386–393. 10.1002/pds.3390 23280590

[B50] McCusker C. Kennedy P. Anderson J. Hicks E. Hanrahan D. (2002). Adjustment in children with intractable epilepsy: Importance of seizure duration and family factors. *Dev. Med. Child Neurol.* 44 681–687. 10.1017/s0012162201002754 12418793

[B51] Moore J. McAuley J. Long L. Bornstein R. (2002). An evaluation of the effects of methylphenidate on outcomes in adult epilepsy patients. *Epilepsy Behav.* 3 92–95. 10.1006/ebeh.2001.0313 12609358

[B52] Mulligan C. Trauner D. (2014). Incidence and behavioral correlates of epileptiform abnormalities in autism spectrum disorders. *J. Autism Dev. Disord.* 44 452–458. 10.1007/s10803-013-1888-6 23872941

[B53] Narr K. Woods R. Lin J. Kim J. Phillips O. Del’Homme M. (2009). Widespread cortical thinning is a robust anatomical marker for attention-deficit/hyperactivity disorder. *J. Am. Acad. Child Adolesc. Psychiatry* 48 1014–1022. 10.1097/CHI.0b013e3181b395c0 19730275 PMC2891193

[B54] Nicolai J. Aldenkamp A. Arends J. Weber J. Vles J. (2006). Cognitive and behavioral effects of nocturnal epileptiform discharges in children with benign childhood epilepsy with centrotemporal spikes. *Epilepsy Behav.* 8 56–70. 10.1016/j.yebeh.2005.08.016 16263335

[B55] Okubo Y. Matsuura M. Asai T. Asai K. Kato M. Kojima T. (1994). Epileptiform EEG discharges in healthy children: Prevalence, emotional and behavioral correlates, and genetic influences. *Epilepsia* 35 832–841. 10.1111/j.1528-1157.1994.tb02520.x 8082631

[B56] Özgen Y. Güngör M. Kutlu M. Kara B. (2021). Clinical and electrophysiological predictors of behavioral disorders in patients with benign childhood epilepsy with centrotemporal spikes. *Epilepsy Behav.* 121:108037. 10.1016/j.yebeh.2021.108037 34058495

[B57] Park J. Choi H. Yum M. Ko T. Shon S. Kim H. (2018). Relationship between aggravation of seizures and methylphenidate treatment in subjects with attention-deficit/hyperactivity disorder and epilepsy. *J. Child Adolesc. Psychopharmacol.* 28 537–546. 10.1089/cap.2017.0070 30089215

[B58] Park K. Ahn H. Yum M. Ko T. Kim H. (2020). Treatment of children and adolescents with epilepsy with atomoxetine. *Psychiatry Investig.* 17 412–416. 10.30773/pi.2019.0287 32295327 PMC7265023

[B59] Pasini A. Paloscia C. Alessandrelli R. Porfirio M. Curatolo P. (2007). Attention and executive functions profile in drug naive ADHD subtypes. *Brain Dev.* 29 400–408. 10.1016/j.braindev.2006.11.010 17207595

[B60] Plihal W. Born J. (1999). Effects of early and late nocturnal sleep on priming and spatial memory. *Psychophysiology* 36 571–582. 10442025

[B61] Prévost J. Lortie A. Nguyen D. Lassonde M. Carmant L. (2006). Nonlesional frontal lobe epilepsy (FLE) of childhood: Clinical presentation, response to treatment and comorbidity. *Epilepsia* 47 2198–2201. 10.1111/j.1528-1167.2006.00714.x 17201725

[B62] Rheims S. Herbillon V. Villeneuve N. Auvin S. Napuri S. Cances C. (2016). ADHD in childhood epilepsy: Clinical determinants of severity and of the response to methylphenidate. *Epilepsia* 57 1069–1077. 10.1111/epi.13420 27237724

[B63] Rosenfeld W. Conry J. Lagae L. Rozentals G. Yang H. Fain R. (2015). Efficacy and safety of perampanel in adolescent patients with drug-resistant partial seizures in three double-blind, placebo-controlled, phase III randomized clinical studies and a combined extension study. *Eur. J. Paediatr. Neurol.* 19 435–445. 10.1016/j.ejpn.2015.02.008 25823975

[B64] Saute R. Dabbs K. Jones J. Jackson D. Seidenberg M. Hermann B. (2014). Brain morphology in children with epilepsy and ADHD. *PLoS One* 9:e95269. 10.1371/journal.pone.0095269 24760032 PMC3997349

[B65] Sayal K. Prasad V. Daley D. Ford T. Coghill D. (2018). ADHD in children and young people: Prevalence, care pathways, and service provision. *Lancet Psychiatry* 5 175–186. 10.1016/S2215-0366(17)30167-0 29033005

[B66] Schreiber J. Lanham D. Trescher W. Sparks S. Wassif C. Caffo B. (2014). Variations in EEG discharges predict ADHD severity within individual Smith-Lemli-Opitz patients. *Neurology* 83 151–159. 10.1212/WNL.0000000000000565 24920862 PMC4117167

[B67] Shewmon D. Erwin R. (1988). Focal spike-induced cerebral dysfunction is related to the after-coming slow wave. *Ann. Neurol.* 23 131–137. 10.1002/ana.410230205 3377436

[B68] Silvestri R. Gagliano A. Calarese T. Aricò I. Cedro C. Condurso R. (2007). Ictal and interictal EEG abnormalities in ADHD children recorded over night by video-polysomnography. *Epilepsy Res.* 75 130–137. 10.1016/j.eplepsyres.2007.05.007 17588723

[B69] Siniatchkin M. Groening K. Moehring J. Moeller F. Boor R. Brodbeck V. (2010). Neuronal networks in children with continuous spikes and waves during slow sleep. *Brain* 133 2798–2813. 10.1093/brain/awq183 20688812

[B70] Socanski D. Aurlien D. Herigstad A. Thomsen P. Larsen T. (2013). Epilepsy in a large cohort of children diagnosed with attention deficit/hyperactivity disorders (ADHD). *Seizure* 22 651–655. 10.1016/j.seizure.2013.04.021 23711613

[B71] Socanski D. Aurlien D. Herigstad A. Thomsen P. Larsen T. (2015). Attention deficit/hyperactivity disorder and interictal epileptiform discharges: It is safe to use methylphenidate? *Seizure* 25 80–83. 10.1016/j.seizure.2015.01.002 25645642

[B72] Stickgold R. Whidbee D. Schirmer B. Patel V. Hobson J. (2000). Visual discrimination task improvement: A multi-step process occurring during sleep. *J. Cogn. Neurosci.* 12 246–254. 10.1162/089892900562075 10771409

[B73] Subchartanan J. Patharathitikul S. Chonchaiya W. (2015). Prevalence of attention deficit hyperactivity disorder in children with epilepsy in a Thai Hospital. *Asian Biomed.* 9 803–807. 10.5372/1905-7415.0906.454

[B74] Torres A. Whitney J. Rao S. Tilley C. Lobel R. Gonzalez-Heydrich J. (2011). Tolerability of atomoxetine for treatment of pediatric attention-deficit/hyperactivity disorder in the context of epilepsy. *Epilepsy Behav.* 20 95–102. 10.1016/j.yebeh.2010.11.002 21146461

[B75] Van Bogaert P. Urbain C. Galer S. Ligot N. Peigneux P. De Tiège X. (2012). Impact of focal interictal epileptiform discharges on behaviour and cognition in children. *Neurophysiol. Clin.* 42 53–58. 10.1016/j.neucli.2011.11.004 22200342

[B76] Verrotti A. Moavero R. Panzarino G. Di Paolantonio C. Rizzo R. Curatolo P. (2018). The challenge of pharmacotherapy in children and adolescents with epilepsy-ADHD comorbidity. *Clin Drug Investig.* 38 1–8. 10.1007/s40261-017-0585-1 29071470

[B77] Volkow N. Fowler J. Wang G. Ding Y. Gatley S. (2002). Mechanism of action of methylphenidate: Insights from PET imaging studies. *J. Atten. Disord.* 6 S31–S43. 10.1177/070674370200601s05 12685517

[B78] Wang M. Zhao Q. Kang H. Zhu S. (2020). Attention deficit hyperactivity disorder (ADHD) in children with epilepsy. *Ir. J. Med. Sci.* 189 305–313. 10.1007/s11845-019-02042-3 31187336

[B79] Wiggs K. Chang Z. Quinn P. Hur K. Gibbons R. Dunn D. (2018). Attention-deficit/hyperactivity disorder medication and seizures. *Neurology* 90 e1104–e1110. 10.1212/WNL.0000000000005213 29476037 PMC5880635

[B80] Yoong M. (2015). Quantifying the deficit-imaging neurobehavioural impairment in childhood epilepsy. *Quant. Imaging Med. Surg.* 5 225–237. 25853081 10.3978/j.issn.2223-4292.2015.01.06 PMC4379313

